# Differential Susceptibility of Two Field *Aedes aegypti* Populations to a Low Infectious Dose of Dengue Virus

**DOI:** 10.1371/journal.pone.0092971

**Published:** 2014-03-24

**Authors:** Arissara Pongsiri, Alongkot Ponlawat, Butsaya Thaisomboonsuk, Richard G. Jarman, Thomas W. Scott, Louis Lambrechts

**Affiliations:** 1 Department of Entomology, Armed Forces Research Institute of Medical Sciences, Bangkok, Thailand; 2 Department of Virology, Armed Forces Research Institute of Medical Sciences, Bangkok, Thailand; 3 Department of Entomology and Nematology, University of California Davis, Davis, California, United States of America; 4 Fogarty International Center, National Institutes of Health, Bethesda, Maryland, United States of America; 5 Insect-Virus Interactions Group, Department of Genomes and Genetics, Institut Pasteur – Centre National de la Recherche Scientifique, Unité de Recherche Associée 3012, Paris, France; University of California Davis, United States of America

## Abstract

**Background:**

The infectious dose required to infect mosquito vectors when they take a blood meal from a viremic person is a critical parameter underlying the probability of dengue virus (DENV) transmission. Because experimental vector competence studies typically examine the proportion of mosquitoes that become infected at intermediate or high DENV infectious doses in the blood meal, the minimum blood meal titer required to infect mosquitoes is poorly documented. Understanding the factors influencing the lower infectiousness threshold is epidemiologically significant because it determines the transmission potential of humans with a low DENV viremia, possibly including inapparent infections, and during the onset and resolution of the viremic period of acutely infected individuals.

**Methodology/Principal Findings:**

We compared the susceptibility of two field-derived *Aedes aegypti* populations from Kamphaeng Phet, Thailand when they were orally exposed to low titers of six DENV-2 isolates derived from the serum of naturally infected humans living in the same region. The infectious dose, time-point post-blood feeding, viral isolate and mosquito population, were significant predictors of the proportion of mosquitoes that became infected. Importantly, the dose-response profile differed significantly between the two *Ae. aegypti* populations. Although both mosquito populations had a similar 50% oral infectious dose (OID_50_), the slope of the dose-response was shallower in one population, resulting in a markedly higher susceptibility at low blood meal titers.

**Conclusions/Significance:**

Our results indicate that mosquitoes in nature vary in their infectious dose-response to DENV. Thus, different mosquito populations have a differential ability to acquire DENV infection at low viremia levels. Future studies on human-to-mosquito DENV transmission should not be limited to OID_50_ values, but rather they should be expanded to account for the shape of the dose-response profile across a range of virus titers.

## Introduction

Recent estimations bring to almost 400 million the number of human dengue virus (DENV) infections that occur worldwide each year, of which about three quarters are expected to be clinically inapparent [1]. Dengue is a self-limiting infection caused by RNA viruses of the genus *Flavivirus* that are transmitted among humans by mosquitoes [2]. DENV include four serotypes (DENV-1, -2, -3 and -4) that are phylogenetically closely related, but antigenically distinct. The principal mosquito vector species of DENV worldwide is *Aedes aegypti*, although *Ae. albopictus* has occasionally been implicated in relatively small-scale dengue outbreaks [3]. Currently, there is no licensed vaccine or commercially available therapeutic for dengue. Disease prevention relies on the control of mosquito vector populations. Transmission occurs when a mosquito bites a human during their viremic period, which typically lasts 4–5 days [4]–[6]. On average, the probability of DENV transmission from symptomatic humans to mosquitoes follows the kinetics of viremia [7], from as soon as two days before the onset of fever to the day following defervescence [8].

One critical parameter underlying a successful human-to-mosquito DENV transmission event is the magnitude of human viremia required to infect mosquitoes. It is well established that the proportion of infected mosquitoes is positively associated with the virus dose ingested in artificial blood meals [9], [10]. The dose-response of mosquitoes generally follows a sigmoid relationship with 0% infection below a lower threshold and 100% infection above an upper threshold. A recent study documented the dose-response of *Ae. aegypti* fed directly on the blood from clinically ill, naturally infected humans in Vietnam [7]. Analysis of the proportion of infected mosquitoes as a function of human plasma viremia confirmed the sigmoid shape of the mosquito dose-response. There was no or little transmission below 10^3^ viral RNA copies/ml of plasma, and close to 100% transmission above 10^9^ viral RNA copies/ml. Estimates for the 50% oral infectious dose (OID_50_), defined as the virus concentration resulting in 50% infected mosquitoes, ranged from 10^6.29^ to 10^7.52^ viral RNA copies/ml of plasma [7]. Interestingly, the OID_50_ differed among serotypes, indicating that viral factors influence the shape of the mosquito dose-response.

Most vector competence studies use blood meal titers that are in the higher part of the mosquito dose-response to DENV (e.g., [11]–[14]). In other words, virus titers in blood meals are typically around or greater than the OID_50_. The lower part of the dose-response (i.e., mosquito infection rates <50%) has generally not been examined, perhaps because vector-virus interaction studies need to maximize the number of infected mosquitoes. Gaining a better understanding of the lower part of the dose-response to DENV is epidemiologically important for at least three reasons. First, epidemic DENV transmission is not always associated with high human viremias [15], [16]. Second, even when peak viremia is high, there are periods of low viremia during the onset and resolution of the viremic period that may contribute to human-to-mosquito DENV transmission. Third, understanding infectiousness to mosquitoes at low viremia levels is important to quantify the potential contribution of people with mild or clinically inapparent infections to overall DENV transmission. Inapparent and mild DENV infections are assumed to be associated with lower viremia levels by extrapolating the positive relationship observed between viremia and disease severity in symptomatic human cases [7], [17]–[19]. The contribution of mild human infections to DENV transmission may be epidemiologically underappreciated because they have not been studied and they tend to outnumber the more severe infections that make up the bulk of the cases detected and studied by public health systems [1], [20], [21].

Here, we investigated the susceptibility of two field-derived *Ae. aegypti* populations that were orally exposed to low infectious doses of six low-passage DENV-2 isolates obtained from human serum samples. All experiments were carried out using the first laboratory-reared generation of mosquitoes derived from wild *Ae. aegypti* collected in Na Bo Kham (NB) and Nakhon Chum (NC), two villages located approximately 25 km apart in Kamphaeng Phet Province, Thailand. Our results indicate that dose-response differed between the two mosquito populations, with one population being more susceptible to DENV infection at low infectious doses despite a similar OID_50_.

## Materials and Methods

### Mosquitoes

Wild *Ae. aegypti* immatures (larvae and pupae) were collected from a variety of artificial containers in several households in Na Bo Kham (NB) and Nakhon Chum (NC) sub-districts, Muang district, Kamphaeng Phet Province, Thailand during December 2011 (experiment 1) and August 2012 (experiment 2). F_0_ adults were allowed to emerge in the laboratory, mate randomly, and feed on defibrinated sheep blood (National Laboratory Animal Center, Mahidol University, Nakhon Pathom, Thailand) through a membrane feeding system. F_1_ eggs were collected on paper towel lining oviposition cups and stored under high humidity. Prior to the experiments, they were hatched synchronously by placing them under low pressure for 30 min. Larvae were reared in 24×34×9 cm plastic trays filled with 2.0 liters of dechlorinated tap water at a density of approximately 200 first instars per tray and fed a standard diet of approximately 1.0 g of fish food pellets (C.P. Hi Pro, Perfect Companion Group Co. Ltd, Bangkok, Thailand) per tray. After emergence, F_1_ adults were housed in plastic 30×30×30 cm cages (Megaview Science Education Service Co. Ltd, Taichung, Taiwan) with permanent access to 10% sucrose. They were maintained under insectary conditions at 28±1°C, 80% humidity, and with a 12∶12 hour light∶dark cycle.

### Virus isolates

Six DENV-2 isolates (designated as 29, 50, 51, 54, 66, and 67) were derived from serum samples obtained between July and September 2010 from people with acute dengue infections in Nakhon Chum (isolates 29, 50, 51), Sa Kaeo (isolate 54) and Na Bo Kham (isolates 66, 67). These villages are located 25–35 km apart in Muang district, Kamphaeng Phet Province, Thailand. The serum samples were obtained as part of larger geographic cluster investigations initiated from ‘index’ cases admitted to the Kamphaeng Phet Provincial Hospital with DENV infections confirmed by RT-PCR. The study site and cluster investigation methodology have been described previously [22]. Virus isolation and identification was performed according to published methods [23]. Phylogenetic analysis based on their complete envelope gene sequence assigned the viruses to the Asian genotype II of DENV-2 (data not shown). Each isolate was passaged four times in *Ae. albopictus* cells (C6/36, ATCC CRL-1660) prior to its use in experimental infections of mosquitoes.

### Ethics statement

The study protocol was approved by the Institutional Review Boards of the Thai Ministry of Public Health, Walter Reed Army Institute of Research, and University of California at Davis. Written informed consent was obtained from study participants and/or parents of participants; assent was obtained from persons more than seven years of age.

### Oral challenge

Experimental infections were conducted as previously described [24]. Briefly, two sets of two-day-old confluent cultures of C6/36 cells in 25 cm^2^ flasks (approximately 10^7^ cells/flask) were inoculated with 1.0 ml of stock virus per flask and incubated at 35°C. Supernatant was harvested five days post-inoculation to prepare the infectious blood meal. The artificial blood meal consisted of 1∶1 mix of defibrinated sheep blood (National Laboratory Animal Center, Mahidol University, Nakhon Pathom, Thailand) and virus suspension. Three infectious doses were prepared by using the viral supernatant undiluted, diluted 0.5 and diluted 0.1 in RPMI 1640 medium with 5% heat-inactivated fetal bovine serum (HIFBS). Four- to seven-day-old *Ae. aegypti* F_1_ females deprived of sucrose and water for 24 hours were offered an infectious blood meal for 30 min through pieces of desalted porcine intestine stretched over water-jacketed glass feeders maintained at 37°C. Samples of the blood meals were saved for subsequent titration by plaque assay. After blood feeding, mosquitoes were briefly sedated with CO_2_ from dry ice and fully engorged females were transferred to clean paper cups. Unfed or partially fed females were discarded. Engorged females were maintained under standard insectary conditions, as described above, and provided cotton soaked with 10% sucrose *ad libitum*.

### Susceptibility

Infection of *Ae. aegypti* midguts by the six DENV-2 isolates was assessed for the three infectious doses at 7 and 14 days post-blood feeding based on the proportion of infected mosquitoes determined by plaque assay. Upon harvest, mosquito bodies were kept individually in 1.0 ml of mosquito diluent (MD), consisting of RPMI 1640 medium with 10% HIFBS with 100 units/ml penicillin and 100 μg/ml streptomycin. Samples were stored at −70°C before processing. They were quickly thawed in a water bath at 35±2°C and homogenized in a mixer mill (Qiagen, Hilden, Germany) at 24 cycles/sec for 2 min. Plaque assay was performed in rhesus monkey kidney cells (LLC-MK_2_, ATCC CCL-7) as previously described [25]. Briefly, the homogenized samples were passed individually through a 0.22 μm syringe filter unit and 0.5 and 0.1 dilutions were prepared in MD. The samples were placed in an ice bath and 100 μl/well were inoculated into a monolayer of LLC-MK_2_ cells in 24-well plates. The virus was adsorbed for 1 hour at room temperature (20–28°C) on a rocker platform. The inoculum was removed and 0.5 ml/well of a first overlay of medium was added. The cells were incubated for 5 days at 35±1°C in a 5±0.5% CO_2_ incubator. The cells were stained with a second overlay of medium containing 4% neutral red (Sigma, St. Louis, USA). Plaques were counted and plaque forming units (PFU)/ml were calculated.

### Data analysis

The study was run in two separate experiments that involved two different sets of DENV isolates and used populations of *Ae. aegypti* that were sampled from the same locations, but on different dates. The isolate and population effects, therefore, were nested within the effect of the experiment. The proportion of mosquitoes that became infected following the infectious blood meal was analyzed with a multifactorial logistic regression that included the effects of experiment, time-point post-blood feeding, infectious dose (log_10_-transformed), mosquito population, viral isolate, and their interactions up to the second-order. An initial analysis with third-order interactions showed that none of the third-order terms had a significant effect. In the models, each mosquito was represented by a single binary value (0 =  uninfected; 1 =  infected) so that individual variation was accounted for. Estimates of odds ratios and their standard errors were obtained with a separate logistic fit of infection status as a function of infectious dose for each combination of time-point, isolate and population. The same logistic fit was used to derive OID_50_ and OID_10_ values and their 95% confidence intervals. All analyses were performed with the software JMP v10.0.2.

## Results

A total of 769 *Ae. aegypti* females were examined for DENV infection by plaque assay, of which 363 (47.2%) were tested at 7 days post-blood feeding (pbf), and 406 (52.8%) at 14 days pbf. The two mosquito populations (referred to as NB and NC here after) were exposed to dilutions of six DENV-2 isolates (experiment 1: isolates 29, 54, 66 and 67; experiment 2: isolates 50 and 51). Among isolates, infectious doses were distributed from 10^3.74^ to 10^5.74^ PFU/ml in each blood meal (median 10^4.74^ PFU/ml). Each combination of mosquito population, virus isolate, and infectious dose was represented by 6–37 mosquitoes (median 19.5). Distribution of sample sizes is given in Table S1. One dose was missing for pairs 54-NB and 67-NB at 7 and 14 days pbf, respectively.

**Figure 1:**
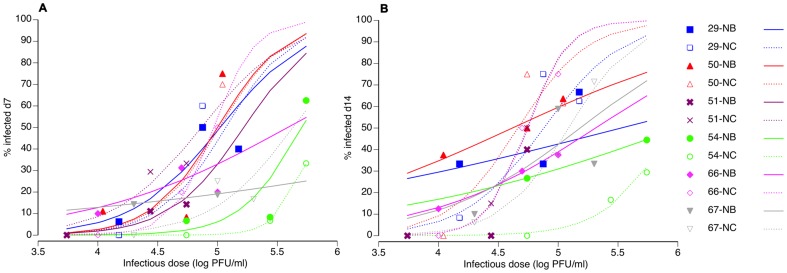
Logistic fits of the observed dose-responses. The percentage of mosquitoes found virus-positive 7 days (**A**) and 14 days (**B**) after oral challenge is shown as a function of the blood meal titer for all combinations of two *Ae. aegypti* populations (NB =  Na Bo Kham; NC =  Nakhon Chum) and six DENV-2 virus isolates (experiment 1: isolates 29, 54, 66 and 67; experiment 2: isolates 50 and 51). Symbols represent empirical data and lines are logistic fits of the data. Solid symbols and lines correspond to the NB population; Open symbols and dashed lines represent the NC population. Note that the logistic fit of the 51-NB pair at day 14 was omitted from the figure because parameter estimates were unstable.

Overall, 76 (20.9%) of the mosquitoes had detectable infections at 7 days pbf, and 129 (31.8%) at 14 days pbf. The difference in the proportion of infected mosquitoes between the two time-points was statistically significant (Table 1). The average viral titer in infected bodies (across infectious doses) was 10^2.41^ PFU/ml at 7 days pbf versus 10^3.02^ PFU/ml at 14 days pbf. The strongest effect influencing the proportion of mosquitoes that became infected following the infectious blood meal was infectious dose (Table 1). In general, the percentage of infected mosquitoes strongly increased with increasing dose (Fig. 1). In addition, there was a statistically significant interaction between the mosquito population and the virus isolate (Table 1), indicating that infection probability was partly determined by the specific pairing of mosquito and virus strains. There was no evidence for a differential effect of mosquito-virus pairs from the same or a different geographical location.

**Table 1:** Multifactorial logistic regression of infection status.

Source	df	L-R χ^2^	*P*-value
Experiment	1	12.3	**0.0005**
Time-point	1	11.5	**0.0007**
Experiment*Time-point	1	0.92	0.3386
Dose	1	61.3	**<0.0001**
Experiment*Dose	1	1.06	0.3041
Time-point*Dose	1	2.62	0.1054
Isolate [within Experiment]	4	22.5	**0.0002**
Population [within Experiment]	2	9.51	**0.0086**
Isolate*Time-point [within Experiment]	4	5.55	0.2352
Isolate*Population [within Experiment]	4	17.2	**0.0018**
Isolate*Dose [within Experiment]	4	2.18	0.7026
Population*Time-point [within Experiment]	2	0.90	0.6390
Population*Dose [within Experiment]	2	11.8	**0.0027**

Significant *P*-values (<0.05) are in bold. df: degrees of freedom; L-R: likelihood ratio.

There was also a highly significant effect of the interaction between the infectious dose and the mosquito population (Table 1). This effect reflected a different shape of the dose-response profile between the two mosquito populations. Across isolates, the dose-response of the NC population was the typical sigmoid relationship with a steep exponential phase. The NB population displayed a shallower dose-response (Fig. 1). The difference was more obvious at 14 (Fig. 1B) than 7 days pbf (Fig. 1A).

**Figure 2:**
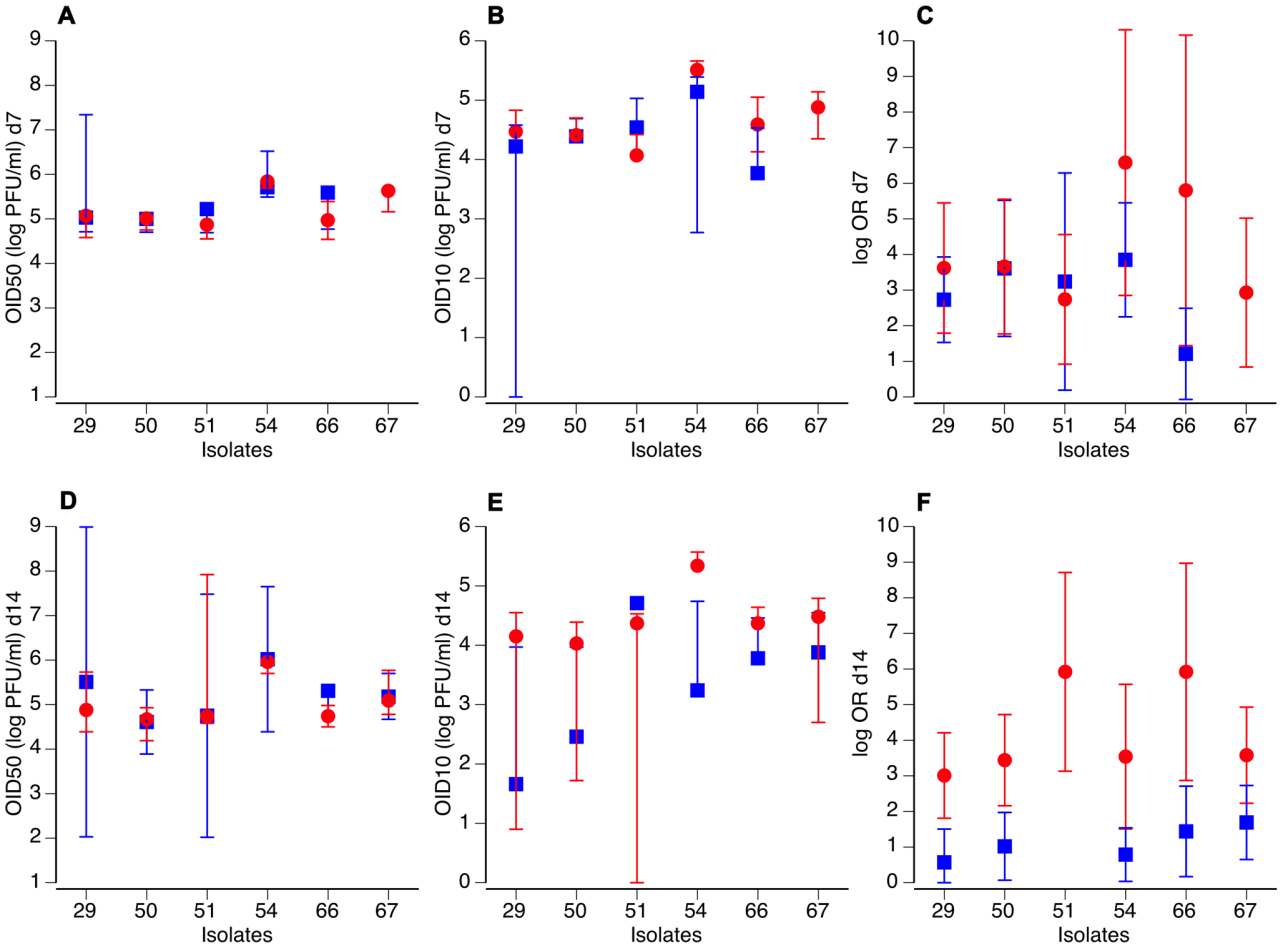
Dose-responses differ between the two *Ae. aegypti* populations. For both *Ae. aegypti* populations (blue squares: Na Bo Kham [NB]; red circles: Nakhon Chum [NC]), OID_50_ values (**A**, **D**), OID_10_ values (**B**, **E**) and estimated odds ratios (**C**, **F**) are shown for each of the six DENV-2 virus isolates. Upper panels (**A**–**C**) correspond to 7 days and lower panels (**D**–**F**) to 14 days after the infectious blood meal. Vertical bars indicate 95% confidence intervals of the OID values and standard errors of the odds ratios. Missing error bars mean that they could not be reliably estimated.

The difference in the overall shape of the dose-response between the two mosquito populations was further analyzed with various metrics derived from the logistic fits of the data. The 50% oral infectious dose (OID_50_) varied among virus isolates, but was almost identical between the two mosquito populations both at 7 (Fig. 2A) and 14 days pbf (Fig. 2D). For most isolates, however, the 10% oral infectious dose (OID_10_) estimates were lower at 14 days pbf for the NB population compared to the NC population (Fig. 2E), supporting the conclusion that the NB population is more susceptible than the NC population to low doses of DENV. This difference was not apparent at 7 days pbf (Fig. 2B). Likewise, the odds ratio of the prevalence-dose relationship was always higher for the NC population at 14 (Fig. 2F), but not 7 days pbf (Fig. 2C). In this case, the odds ratio describes the relative increase in prevalence for a one-unit increase in infectious dose. Higher odds ratio reflect the stronger correlation (i.e., steeper sigmoid) between the proportion of infected mosquitoes and the blood meal titer for the NC population than for the NB population.

## Discussion

We carried out experimental infections of mosquitoes at the lower part of their dose-response to DENV. A recent estimate of DENV-2 OID_50_ measured 12 days after the infectious blood meal in *Ae. aegypti* was 10^6.29^ viral RNA copies/ml of plasma [7]. Based on their calibration of the quantitative RT-PCR assay used to measure plasma viremia levels, Nguyen *et al*. [7] estimated that 10^6.29^ DENV-2 genome copies/ml were equivalent to 10^4.43^ PFU/ml of plasma. Blood meal titers in our experiments ranged from 10^3.74^ to 10^5.74^ PFU/ml of blood, thus bracketing the previously reported OID_50_ value for DENV-2. Accordingly, we observed an infection prevalence ranging from 0% to 75% among population-isolate-dose combinations (Fig. 1). We chose to use plaque assay rather than a more sensitive quantitative RT-PCR assay because viral RNA concentration does not directly translate into infectious titer [26]. Fourteen days after the infectious blood meal, virus in the bodies of mosquitoes with an established DENV infection is expected to have reached infectious titers largely exceeding the plaque assay detection threshold (10 PFU/ml).

Our results support the idea that wild type *Ae. aegypti* vary in their infectious dose-response to oral DENV challenge. Because environmental conditions were controlled in our experiments, we interpret phenotypic differences observed between NB and NC populations as genetic. A previous study reported that *Ae. aegypti* populations sampled within 25 km of each other in Thailand, including the Kamphaeng Phet area, were genetically differentiated [27]. Elucidating the genetic factors responsible for the higher susceptibility of the NB population at a low infectious dose compared to the NC population may provide greater insight into the genetic basis of vector competence. Although there was considerable variation in infection prevalence among the six low-passage DENV-2 isolates (OID_50_ values ranged from 10^4.61^ to 10^6.02^ PFU/ml at 14 days pbf), we observed that the overall shape of the dose-response profile of both *Ae. aegypti* populations was consistent across isolates. In particular, odds ratios of the prevalence-dose relationship estimated at 14 days pbf were always higher for the NC population than for the NB population (Fig. 2F), as illustrated by the steeper slope of the central part of the sigmoid relationship. This indicates that the overall shape of the dose-response is an intrinsic feature of the mosquito population that transcends specific interactions that have been reported to occur between mosquito and virus genotypes [28].

We examined only two *Ae. aegypti* populations and a limited set of DENV-2 isolates of the Asian genotype II. Additional studies are necessary to determine whether our conclusions can be extrapolated to other mosquito populations and virus genotypes. Here, we provide proof-of-principle that variation in dose-response may exist among mosquito populations and should be considered in the design of future studies. Although individual dose-responses curves of some population-isolate pairs lacked accuracy because of relatively small sample sizes (Table S1), the interaction between mosquito population and infectious dose was statistically significant across population-isolate pairs. In other words, the overall effect was robustly supported by larger sample sizes than those obtained for each population-isolate pair.

Differences in the ability of vector populations to acquire DENV infection at low blood meal titers may help to explain some puzzling aspects of dengue epidemiology. For example, this process may have contributed to rare instances of epidemic DENV transmission associated with low viremia in humans [15], [16]. Variation in the ability of mosquito populations to acquire DENV infection at low viremia levels also may result in heterogeneities in the potential contribution of people with mild dengue infections to overall DENV transmission. Assuming that inapparent/mild DENV infections are associated with lower viremias [7], [17]–[19], their epidemiological significance may depend on the local mosquito population with which they interact.

A caveat of our study is that we focused on mosquito infection prevalence and did not consider virus dissemination from the midgut to other tissues and, thus, the potential for transmission (i.e., vector competence). Because the blood meal titers were relatively low, the numbers of mosquitoes that developed a disseminated infection in head tissues (data not shown) were too small for a meaningful analysis. Infection of the mosquito midgut is a critical prerequisite for transmission via mosquito bite [29]. Whether our conclusions hold for actual mosquito-to-human DENV transmission requires additional investigation. When possible, in future studies we recommend that investigators try to move away from artificial mosquito feedings methods by including more natural conditions of DENV transmission [30]. A recent study conducted in Vietnam [7] has set a higher standard for human-to-mosquito transmission experiments and highlighted the shortcomings of artificial blood meals. For example, DENV-reactive antibody levels in human plasma samples were negatively correlated with the probability of virus transmission by mosquitoes, independently of viremia level [7]. Such immunological factors are not accounted for in artificial infectious blood meals.

One important implication of our results is that a single summary metric, such as the OID_50_, is not sufficient to describe the complexity of mosquito-virus interactions that underlies mosquito susceptibility to DENV infection and, perhaps, mosquito-virus interactions in general. Indeed, *Ae. aegypti* populations in NB and NC had similar OID_50_ values, yet their susceptibilities to DENV at a low infectious dose were significantly different. We recommend, therefore, that in the future investigators studying human-to-mosquito DENV transmission consider the entire infectious dose-response profile.

## Supporting Information

Table S1 **Sample sizes.** For each combination of mosquito population, virus isolate and infectious dose, the number of individual mosquitoes assayed (N) is indicated.
(DOC)

## References

[1] Bhatt S, Gething PW, Brady OJ, Messina JP, Farlow AW, et al (2013) The global distribution and burden of dengue. Nature 496: 504–507. 23563266 10.1038/nature12060 PMC3651993

[2] Simmons CP, Farrar JJ, Nguyen VV, Wills B (2012) Dengue. N Engl J Med 366: 1423–1432. 22494122 10.1056/NEJMra1110265

[3] Lambrechts L, Scott TW, Gubler DJ (2010) Consequences of the expanding global distribution of *Aedes albopictus* for dengue virus transmission. PLoS Negl Trop Dis 4: e646. 20520794 10.1371/journal.pntd.0000646 PMC2876112

[4] Gubler DJ, Suharyono W, Tan R, Abidin M, Sie A (1981) Viraemia in patients with naturally acquired dengue infection. Bull World Health Organ 59: 623–630. 6976230 PMC2396101

[5] Siler JF, Hall MW, Hitchens AP (1926) Dengue: its history, epidemiology, mechanism of transmission, etiology, clinical manifestations, immunity, and prevention. Philippine J Sci 29: 1–302.

[6] Simmons JS, St. John JH, Reynolds FHK (1931) Experimental studies of dengue. Philippine J Sci 44: 1–247.

[7] Nguyen N, Thi Hue Kien D, Tuan T, Quyen N, Tran C, et al (2013) Host and viral features of human dengue cases shape the population of infected and infectious *Aedes aegypti* mosquitoes. Proc Natl Acad Sci U S A 110: 9072–9077. 23674683 10.1073/pnas.1303395110 PMC3670336

[8] Nishiura H, Halstead SB (2007) Natural history of dengue virus (DENV)-1 and DENV-4 infections: reanalysis of classic studies. J Infect Dis 195: 1007–1013. 17330791 10.1086/511825

[9] Bennett KE, Olson KE, de Lourdes Munoz M, Fernandez-Salas I, Farfan-Ale JA, et al (2002) Variation in vector competence for dengue 2 virus among 24 collections of *Aedes aegypti* from Mexico and the United States. Am J Trop Med Hyg 67: 85–92. 12363070 10.4269/ajtmh.2002.67.85

[10] Vazeille-Falcoz M, Mousson L, Rodhain F, Chungue E, Failloux AB (1999) Variation in oral susceptibility to dengue type 2 virus of populations of *Aedes aegypti* from the islands of Tahiti and Moorea, French Polynesia. Am J Trop Med Hyg 60: 292–299. 10072154 10.4269/ajtmh.1999.60.292

[11] Armstrong PM, Rico-Hesse R (2001) Differential susceptibility of *Aedes aegypti* to infection by the American and Southeast Asian genotypes of dengue type 2 virus. Vector Borne Zoonotic Dis 1: 159–168. 12680353 10.1089/153036601316977769 PMC3057097

[12] Gubler DJ, Nalim S, Tan R, Saipan H, Sulianti Saroso J (1979) Variation in susceptibility to oral infection with dengue viruses among geographic strains of *Aedes aegypti* . Am J Trop Med Hyg 28: 1045–1052. 507282 10.4269/ajtmh.1979.28.1045

[13] Hanley KA, Nelson JT, Schirtzinger EE, Whitehead SS, Hanson CT (2008) Superior infectivity for mosquito vectors contributes to competitive displacement among strains of dengue virus. BMC Ecol 8: 1. 18269771 10.1186/1472-6785-8-1 PMC2263032

[14] Sanchez-Vargas I, Scott JC, Poole-Smith BK, Franz AW, Barbosa-Solomieu V, et al (2009) Dengue virus type 2 infections of *Aedes aegypti* are modulated by the mosquito's RNA interference pathway. PLoS Pathog 5: e1000299. 19214215 10.1371/journal.ppat.1000299 PMC2633610

[15] Gubler DJ, Reed D, Rosen L, Hitchcock JR Jr (1978) Epidemiologic, clinical, and virologic observations on dengue in the Kingdom of Tonga. Am J Trop Med Hyg 27: 581–589. 677371 10.4269/ajtmh.1978.27.581

[16] Gubler DJ, Suharyono W, Lubis I, Eram S, Gunarso S (1981) Epidemic dengue 3 in central Java, associated with low viremia in man. Am J Trop Med Hyg 30: 1094–1099. 7283006 10.4269/ajtmh.1981.30.1094

[17] Libraty DH, Endy TP, Houng HS, Green S, Kalayanarooj S, et al (2002) Differing influences of virus burden and immune activation on disease severity in secondary dengue-3 virus infections. J Infect Dis 185: 1213–1221. 12001037 10.1086/340365

[18] Murgue B, Roche C, Chungue E, Deparis X (2000) Prospective study of the duration and magnitude of viraemia in children hospitalised during the 1996-1997 dengue-2 outbreak in French Polynesia. J Med Virol 60: 432–438. 10686027 10.1002/(sici)1096-9071(200004)60:4<432::aid-jmv11>3.0.co;2-7

[19] Vaughn DW, Green S, Kalayanarooj S, Innis BL, Nimmannitya S, et al (2000) Dengue viremia titer, antibody response pattern, and virus serotype correlate with disease severity. J Infect Dis 181: 2–9. 10608744 10.1086/315215

[20] Endy TP, Anderson KB, Nisalak A, Yoon IK, Green S, et al (2011) Determinants of inapparent and symptomatic dengue infection in a prospective study of primary school children in Kamphaeng Phet, Thailand. PLoS Negl Trop Dis 5: e975. 21390158 10.1371/journal.pntd.0000975 PMC3046956

[21] Yoon IK, Rothman AL, Tannitisupawong D, Srikiatkhachorn A, Jarman RG, et al (2012) Underrecognized mildly symptomatic viremic dengue virus infections in rural thai schools and villages. J Infect Dis 206: 389–398. 22615312 10.1093/infdis/jis357 PMC3490697

[22] Mammen MP, Pimgate C, Koenraadt CJ, Rothman AL, Aldstadt J, et al (2008) Spatial and temporal clustering of dengue virus transmission in Thai villages. PLoS Med 5: e205. 18986209 10.1371/journal.pmed.0050205 PMC2577695

[23] Klungthong C, Gibbons RV, Thaisomboonsuk B, Nisalak A, Kalayanarooj S, et al (2007) Dengue virus detection using whole blood for reverse transcriptase PCR and virus isolation. J Clin Microbiol 45: 2480–2485. 17522268 10.1128/JCM.00305-07 PMC1951229

[24] Lambrechts L, Fansiri T, Pongsiri A, Thaisomboonsuk B, Klungthong C, et al (2012) Dengue-1 virus clade replacement in Thailand associated with enhanced mosquito transmission. J Virol 86: 1853–1861. 22130539 10.1128/JVI.06458-11 PMC3264336

[25] Thomas SJ, Nisalak A, Anderson KB, Libraty DH, Kalayanarooj S, et al (2009) Dengue plaque reduction neutralization test (PRNT) in primary and secondary dengue virus infections: How alterations in assay conditions impact performance. Am J Trop Med Hyg 81: 825–833. 19861618 10.4269/ajtmh.2009.08-0625 PMC2835862

[26] Choy MM, Ellis BR, Ellis EM, Gubler DJ (2013) Comparison of the mosquito inoculation technique and quantitative real time polymerase chain reaction to measure dengue virus concentration. Am J Trop Med Hyg 89: 1001–1005. 24019432 10.4269/ajtmh.13-0100 PMC3820311

[27] Bosio CF, Harrington LC, Jones JW, Sithiprasasna R, Norris DE, et al (2005) Genetic structure of *Aedes aegypti* populations in Thailand using mitochondrial DNA. Am J Trop Med Hyg 72: 434–442. 15827282

[28] Fansiri T, Fontaine A, Diancourt L, Caro V, Thaisomboonsuk B, et al (2013) Genetic mapping of specific interactions between *Aedes aegypti* mosquitoes and dengue viruses. PLoS Genet 9: e1003621. 23935524 10.1371/journal.pgen.1003621 PMC3731226

[29] Black WC, Bennett KE, Gorrochotegui-Escalante N, Barillas-Mury CV, Fernandez-Salas I, et al (2002) Flavivirus susceptibility in *Aedes aegypti* . Arch Med Res 33: 379–388. 12234528 10.1016/s0188-4409(02)00373-9

[30] Lambrechts L, Failloux AB (2012) Vector biology prospects in dengue research. Mem Inst Oswaldo Cruz 107: 1080–1082. 23295765 10.1590/s0074-02762012000800022

