# Supplementary material for: Differential Susceptibility of Two Field Aedes aegypti Populations to a Low Infectious Dose of Dengue Virus
Source: PLoS One. 2014 Mar 24;9(3):e92971. doi: 10.1371/journal.pone.0092971 (PMC3963970; doi:10.1371/journal.pone.0092971)
Supplement: Table S1 — Sample sizes. For each combination of mosquito population, virus isolate and infectious dose, the number of individual mosquitoes assayed (N) is indicated. (DOC) [file pone.0092971.s001.doc]

| Experiment | Isolate | Population | Dilution | N (d7) | N (d14) | N total |
| --- | --- | --- | --- | --- | --- | --- |
| 1 | 29 | NB | 0.1 | 16 | 18 | 34 |
|  |  |  | 0.5 | 14 | 12 | 26 |
|  |  |  | 1 | 5 | 3 | 8 |
|  |  | NC | 0.1 | 11 | 12 | 23 |
|  |  |  | 0.5 | 5 | 4 | 9 |
|  |  |  | 1 | 5 | 8 | 13 |
|  | 54 | NB | 0.1 | 15 | 15 | 30 |
|  |  |  | 0.5 | 12 | 0 | 12 |
|  |  |  | 1 | 16 | 18 | 34 |
|  |  | NC | 0.1 | 12 | 12 | 24 |
|  |  |  | 0.5 | 15 | 18 | 33 |
|  |  |  | 1 | 15 | 17 | 32 |
|  | 66 | NB | 0.1 | 10 | 8 | 18 |
|  |  |  | 0.5 | 16 | 20 | 36 |
|  |  |  | 1 | 5 | 8 | 13 |
|  |  | NC | 0.1 | 11 | 10 | 21 |
|  |  |  | 0.5 | 5 | 6 | 11 |
|  |  |  | 1 | 2 | 4 | 6 |
|  | 67 | NB | 0.1 | 7 | 10 | 17 |
|  |  |  | 0.5 | 16 | 17 | 33 |
|  |  |  | 1 | 0 | 9 | 9 |
|  |  | NC | 0.1 | 14 | 16 | 30 |
|  |  |  | 0.5 | 8 | 8 | 16 |
|  |  |  | 1 | 6 | 7 | 13 |
| 2 | 50 | NB | 0.1 | 9 | 8 | 17 |
|  |  |  | 0.5 | 12 | 14 | 26 |
|  |  |  | 1 | 8 | 11 | 19 |
|  |  | NC | 0.1 | 9 | 9 | 18 |
|  |  |  | 0.5 | 13 | 16 | 29 |
|  |  |  | 1 | 10 | 13 | 23 |
|  | 51 | NB | 0.1 | 12 | 16 | 28 |
|  |  |  | 0.5 | 9 | 11 | 20 |
|  |  |  | 1 | 7 | 10 | 17 |
|  |  | NC | 0.1 | 7 | 8 | 15 |
|  |  |  | 0.5 | 17 | 20 | 37 |
|  |  |  | 1 | 9 | 10 | 19 |

NB=Na Bo Kham; NC=Nakhon Chum.
